# Carcinoïde primitif du rein métastasant après 12 ans

**DOI:** 10.11604/pamj.2016.23.76.9033

**Published:** 2016-03-10

**Authors:** Dhouha Bacha, Ahlem Lahmar, Lassad Gharbi, Sana Ben Slama, Saadia Bouraoui, Samia Chatti, Sabeh Mzabi Regaya

**Affiliations:** 1 Service d'Anatomie et de Cytologie Pathologiques, Hôpital Mongi Slim, La Marsa, Tunisie; 2 Service de Chirurgie Générale, Hôpital Mongi slim, La Marsa, Tunisie; 3 Service d'Anatomie et de Cytologie Pathologiques, Clinique International Hannibal, Tunisie

**Keywords:** Tumeur carcinoïde, rein, métastase, neuroendocrine, Carcinoid tumors, kidney, metastasis, neuroendocrine

## Abstract

Les carcinoïdes primitifs du rein sont rares avec une centaine de cas rapportés dans la littérature. Sur le plan histologique, il s'agit d'une tumeur bien différenciée dont la morphologie rejoint souvent celle des carcinoïdes dans les autres localisations. Nous rapportons un cas de carcinoïde primitif du rein survenant chez un homme de 41 ans, découvert à la suite de métastases hépatiques. La tumeur était particulière par son architecture tubulo-papillaire, suggérant à tort le diagnostic de carcinome papillaire du rein. Ce diagnostic a été redressé 12 ans après, à la suite de l'apparition d'autres métastases hépatiques, osseuses et pulmonaires.

## Abstract (en)

Primary renal cancinoids are rare, with one hundred cases reported in the literature. Histologically, it is a well-differentiated tumor which shares a morphological pattern similar to that seen in carcinoids identified at other anatomic locations. We report a case of a 45-year-old man with primitive renal carcinoid, with diagnosis made after the detection of liver metastases. The tumour was peculiar given its tubulopapillary architecture, wrongly suggesting a diagnosis of papillary renal carcinoma. This diagnosis was revised 12 years after, following the appearance of other liver, bone and lung metastases.

## Introduction

Les carcinoïdes sont des tumeurs neuroendocrines (TNE) bien différenciées, qui siègent le plus souvent au niveau gastro-intestinal et broncho-pulmonaire [1]. La localisation rénale est rare avec environ 100 cas rapportés dans la littérature[2]. Le diagnostic est habituellement confirmé à l’étude histologique, aidée par l'analyse immuno-histochimique. Du fait de la rareté cette tumeur, ce diagnostic est parfois difficile. Nous rapportons un cas de carcinoïde primitif du rein découvert à la suite de métastases hépatiques.

## Patient et observation

**Figure 1:**
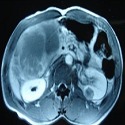
IRM abdominale: volumineuse lésion kystique multi cloisonnée du foie. Nodule de la lèvre postérieure du rein, se rehaussant de manière hétérogène après injection du Gadol

Patient âgé de 41 ans, hospitalisé pour l'exploration d'une double localisation tumorale hépatique et rénale, découverte à la suite de douleurs de l'hypochondre droit. L'examen clinique trouvait une masse sous costale droite sensible à la palpation. La tomodensitométrie (TDM) abdominale montrait que la masse palpée était au dépend des segments VI et VII du foie. Elle mesurait 95 mm de diamètre et était hypo dense, arrondie avec des limites nettes. Elle ne prenait pas le produit de contraste. Cet aspect évoquait un kyste hydatique du foie. Au niveau du rein gauche, l'examen trouvait une formation tissulaire de la lèvre postérieure, prenant le contraste et mesurant 30 mm de diamètre. L'imagerie par résonnance magnétique (IRM) montrait que le nodule rénal était en hypo signal T1 et en hyper signal T2, se rehaussant de manière hétérogène. La masse hépatique était multi-cloisonnée, en hyper signal T2 hétérogène. Les cloisons étaient épaisses et bourgeonnantes par endroits, prenant le contraste sur les séquences tardives (Figure 1).

**Figure 2:**
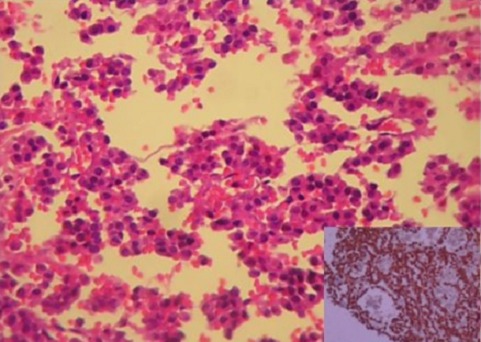
Biopsie hépatique: prolifération tumorale d'architecture pseudo-papillaire et tubulaire (hématoxyline Eosine x 200). En cartouche: immuno-marquage dense et diffus des cellules tumorales avec la synaptophysine (IHC x 100)

Le diagnostic de kyste hydatique hépatique devenait peu probable en dehors d'une forme atypique. Les diagnostics évoqués étaient soit une métastase hépatique d'une tumeur rénale ou une association fortuite d'un cystadénome hépatique et d'une tumeur rénale. Biologiquement, la sérologie du kyste hydatique et de l’échinococcose alvéolaire était négative. Les marqueurs tumoraux (œfoeto-protéine,CA19-9 et antigène carcino-embryonnaire) étaient négatifs. Il a été décidé de réaliser une résection des lésions hépatiques avec une tumorectomie rénale. Sur la pièce de résection hépatique, la tumeur mesurait 12 x 4 cm, était d'aspect pseudo-kystique avec un nodule charnu blanc jaunâtre de 4 cm de grand axe. Le rein montrait un nodule ferme de 2,5 x 2 cm, d'aspect hétérogène jaune-grisâtre avec des remaniements hémorragiques. L'examen histologique le la tumeur hépatique montrait une prolifération d'architecture pseudo-papillaire et tubulaire au sein d'un stroma richement vascularisé (Figure 2). Les cellules tumorales étaient cubiques, à noyaux réguliers avec une chromatine granulaire. L’étude immuno-histochimique (IHC) montrait la positivité de la cytokératine totale et de la vimentine. La chromogranine A était négative. L'examen histologique et IHC de la tumeur rénale était comparable à celui observé au niveau du foie. L′index mitotique était évalué à 3 mitoses /10 champs au fort grossissement (CFG). Il n'a pas été observé de foyers de nécrose tumorale. L'index de prolifération, évalué à l’étude IHC par le Ki 67 était d'environ 3% des cellules tumorales. Les limites chirurgicales hépatiques et rénales passaient en zone saine.

Le diagnostic retenu était celui d'un carcinome papillaire du rein de type I, de grade I de Fürhman métastasant au niveau du foie. Les suites opératoires étaient simples et le patient était perdu de vue. Douze ans après, il avait consulté pour l'apparition d'autres métastases hépatiques, osseuses et pulmonaires. La biopsie hépatique montrait une prolifération tumorale tubulaire d'aspect comparable à celui observé dans le foie et le rein. A l’étude IHC, les cellules tumorales étaient diffusément positives avec la cytokératine totale, la synaptophysine et le CD 56 (Figure 2). Elles étaient négatives avec la chromogranine A. Au vue de ces résultats, le diagnostic final était celui d'un carcinoïde primitif du rein associé à de multiples localisations secondaires hépatiques, osseuses et pulmonaires. Le patient a reçu une chimiothérapie à base de Cisplatine. Après 18 mois d’évolution, le malade est stable.

## Discussion

Les carcinoïdes sont des tumeurs neuroendocrines (TNE) bien différenciées, qui siègent le plus souvent au niveau gastro-intestinal et broncho-pulmonaire [1]. La localisation rénale est rare avec environ 100 cas rapportés dans la littérature. La classification de l'organisation mondiale de la santé (OMS) avait subdivisé les TNE du rein en carcinoïdes, qui sont bien différenciés et en carcinomes neuro-endocrines, qui sont peu différenciés [2]. En 2013, la société internationale de pathologie urologique avait proposé une modification de cette classification, subdivisant les TNE urologiques en bas et haut grade [3]. Les carcinoïdes du rein se développent préférentiellement sur une uropathie malformative dont la plus fréquente est représentée par les reins en fer à cheval, ou sur d'autres anomalies comme les reins polykystiques ou les tératomes matures [4, 5].

Cliniquement, les douleurs abdominals et lombaires représentent le motif de consultation le plus fréquent. Un syndrome carcinoïde est rare, survenant dans moins de 10% des cas. La tumeur peut être de découverte fortuite dans 25% des cas [5, 6]. Dans le cas présenté, la tumeur rénale était découverte après l'apparition de métastases hépatiques, responsables de douleurs de l'hypochondre droit. Sur le plan radiologique, la TDM et l'IRM abdominales ne permettent pas une distinction nette entre un carcinoïde et un carcinome à cellules rénales. La TDM montre une masse solide bien limitée, hétérogène, avec présence possible d'un contingent kystique et de calcifications [7, 5]. La scintigraphie aux récepteurs à la somatostatine et la tomographie par émission de positon (PET Scan) apportent un intérêt pour le bilan d'extension et la surveillance après traitement chirurgical [8].

Sur le plan macroscopique, il s'agit d'une tumeur unique, bien limitée, d'aspect lobulé. La présence d'une hémorragie focale, de calcifications et de remaniements kystiques est habituelle. La nécrose est rare[9]. L'examen histologique et l’étude IHC sont comparables aux carcinoïdes des autres localisations. La tumeur est disposée en nappes, cordons ou en travées au sein d'un stroma richement vascularisé. Les cellules tumorales sont monomorphes, de taille moyenne à grande avec un cytoplasme abondant éosinophile. Le pléomorphisme nucléaire est souvent modéré. L'activité mitotique est habituellement faible, moins de 2 mitoses/10 CFG. L'atteinte de la veine rénale, l'infiltration de la graisse péri-rénale et la découverte de métastases ganglionnaires au moment du diagnostic, sont possibles [3]. Sur le plan IHC, les cellules tumorales des carcinoïdes sont marquées avec les anticorps anti-pancytokératines et EMA et par les marqueurs neuro-endocrines (chromogranine A, synaptophysine et CD56. Les anticorps anti-CK7 et CK20 sont souvent négatifs [9].

Dans notre cas, l'architecture était tubulaire et pseudo-papillaire, ce qui est rarement observé dans les carcinoïdes et dans les tumeurs neuro-endocrines en général. Cet aspect était à l'origine de l'erreur du diagnostic initial de carcinome papillaire du rein d'autant plus que les cellules étaient négatives avec la chromogranine A. Dans les carcinoïdes du rein, l'architecture pseudo-papillaire est rare et il ne s'agit pas de vraies papilles munies d'axes conjonctivo-vasculaires bien individualisés. De plus, la vascularisation est plus abondante que dans les carcinomes papillaires. Sur le plan IHC, la cytokératine 7 est exprimée dans plus de 80% des carcinomes papillaires [3, 9]. Dans notre cas, l'absence de positivité des cellules tumorales avec la chromogranine A pourrait s'expliquer par sa sensibilité relativement moins bonne que celle des autres marqueurs neuro-endocrines, notamment la synaptophysine [3]. Après le diagnostic d'une tumeur carcinoïde du rein, il est impératif d’éliminer une origine digestive ou pulmonaire par un bilan complet d'imagerie. La pathogénie des carcinoïdes du rein est sujet de controverses puisqu’à l’état normal, le parenchyme rénal est dépourvu de cellules neuro-endocrines. Pour certains auteurs, les anomalies malformatives, congénitales ou acquises qui s'associent souvent aux carcinoïdes du rein s'accompagneraient d'un état inflammatoire chronique du parenchyme sous- jacent. Ceci entrainerait des lésions de métaplasie intestinale de l'urothélium tapissant les cavités pyélo-calicielles, avec possible transformation en cellules neuro-endocrines qui s'interposeraient entre les cellules intestinales [5, 6].

Le traitement des carcinoïdes du rein consiste en une néphrectomie radicale ou partielle associée à curage ganglionnaire et si possible une métastasectomie. Les analogues à la somatostatine et la thérapie à base d'Octréotide seraient plus efficaces que la chimiothérapie [6]. Dans notre cas, le patient a initialement bénéficié d'une tumorectomie rénale et hépatique. A l'apparition secondaire des métastases osseuses et pulmonaires,une chimiothérapie à base de Cisplatine a permis la stabilisation tumorale après un recul de 18 mois. Le pronostic des carcinoïdes du rein est difficile à prévoir du fait de la rareté des cas rapportés et l'absence jusqu’à ce jour de classification validée pour les tumeurs neuro-endocrines urologiques. Certains auteurs proposent d'appliquer les classifications des TNE pulmonaires et digestives. Ces classifications se basent sur la présence ou l'absence de nécrose, l'index mitotique et l'index de prolifération Ki67 [2, 10]. Dans notre cas, il s'agirait respectivement d'un carcinoïde atypique et d'une TNE de Grade 2. Le suivi clinique, biologique et radiologique doit être prolongé. La scintigraphie à l'Octréotide et le PET Scan sont plus performants que les méthodes d'imagerie classique pour la détection de métastases ou de récidives même de petite taille [7, 8].

## Conclusion

Le carcinoïde primitif du rein est rare. Son diagnostic est confirmé à l'histologie, aidée par l'IHC moyennant au moins trois marqueurs neuro-endocrines. Une architecture tumorale tubulo-papillaire est possible. L'utilisation des classifications pronostiques des TNE bronchiques et digestives permettrait l’évaluation du comportement évolutif de ces tumeurs. L'apparition possible de métastases tardives nécessiterait un suivi prolongé.
